# Investigation of Cytotoxicity, Permeability, and Stability of Captopril-Loaded Chitosan-Ascorbate Buccal Films

**DOI:** 10.3390/ph19071058

**Published:** 2026-07-08

**Authors:** Hala Rayya, Raghad Alsheikh, Dániel Nemes, Lajos Nagy, Géza Regdon, Ildikó Bácskay, Katalin Kristó, Krisztián Pamlényi

**Affiliations:** 1 Institute of Pharmaceutical Technology and Regulatory Affairs, University of Szeged, Eötvös u. 6., H-6720 Szeged, Hungary; rayya.hala@stud.u-szeged.hu (H.R.); pamlenyi.krisztian@szte.hu (K.P.); 2 Department of Pharmaceutical Technology, Faculty of Pharmacy, University of Debrecen, H-4002 Debrecen, Hungarynemes.daniel@pharm.unideb.hu (D.N.); bacskay.ildiko@pharm.unideb.hu (I.B.); 3 Doctorate School of Pharmaceutical Sciences, University of Debrecen, H-4002 Debrecen, Hungary; 4 Department of Applied Chemistry, Institute of Chemistry, Faculty of Science and Technology, University of Debrecen, H-4032 Debrecen, Hungary; 5 Institute of Healthcare Industry, University of Debrecen, H-4032 Debrecen, Hungary

**Keywords:** buccal mucoadhesive films, captopril, chitosan-ascorbate, cytotoxicity, permeability, stability, TR146 cells

## Abstract

**Background**: Buccal films have attracted continued interest due to their several advantages. However, developing effective buccal films for hydrophilic drugs remains challenging due to limited buccal permeability. Captopril, a water-soluble ACEI, represents a suitable model for evaluating buccal films. Chitosan–ascorbate has previously been studied in solution for its cytotoxicity and permeation-enhancing properties. However, its performance as a buccal-film dosage form requires further investigation. The present study extends earlier formulation work by evaluating the in vitro cytotoxicity, permeability, and stability of captopril-loaded chitosan–ascorbate buccal films. **Methods**: Blank and captopril-loaded films were assessed. Cytotoxicity and permeability were evaluated using TR146 cells and compared with chitosan–acetate films as a reference. Accelerated stability test (40 °C/75% RH, three months) was performed to monitor moisture content, mechanical properties, and drug content. FT-IR spectroscopy was used to investigate potential chemical interactions. **Results**: All films maintained cell viability above 80%. Chitosan–ascorbate films significantly increased captopril permeation compared with chitosan–acetate films, achieving up to a 12–16-fold increase in cumulative drug permeation. The stability test did not reveal any new chemical reactions or interactions based on FT-IR analysis, indicating that the polymer system remained structurally stable; however, it showed continuous moisture uptake, slight deterioration of mechanical properties and a decrease in drug content. **Conclusions**: This study provides an in vitro, dosage-form–level evaluation of captopril-loaded chitosan–ascorbate buccal films, demonstrating acceptable cytocompatibility and moderate enhancement of permeability. However, significant moisture sensitivity under accelerated conditions can represent limitations, highlighting the importance of protective packaging or further formulation optimization to overcome this.

## 1. Introduction

The buccal route has increasingly attracted attention in pharmaceutical research and development as a valuable alternative for both local and systemic drug delivery [1,2]. Unlike the oral route, buccal administration bypasses the gastrointestinal tract and hepatic first-pass metabolism, potentially improving bioavailability and the onset of action while minimizing drug degradation in the acidic environment of the stomach [3,4]. This is particularly beneficial for drugs with limited oral bioavailability, pH sensitivity, or short half-lives [5].

Among the different dosage forms used for buccal drug delivery, mucoadhesive films represent an innovative and patient-friendly system, especially suited for pediatric, geriatric and dysphagic populations [6,7]. These thin polymeric films are designed to adhere to the buccal mucosa, allowing fast and controlled drug release at the site of absorption. Compared to other buccal dosage forms such as tablets, films offer advantages including greater flexibility, uniform drug distribution, easy application, and reduced risk of accidental swallowing [6,8].

Despite the advantages of buccal administration, the oral mucosa presents a permeability barrier, particularly for hydrophilic and ionized compounds [9]. Drug transport across the buccal epithelium occurs via transcellular and paracellular pathways, and permeability is strongly influenced by molecular size, lipophilicity, and ionization state [10]. Hydrophilic molecules often exhibit limited passive diffusion across the lipid-rich epithelial barrier, which may restrict systemic absorption [9].

Captopril (CAP), a hydrophilic angiotensin-converting enzyme inhibitor (ACEI), is a well-known antihypertensive agent used in the treatment of hypertension, congestive heart failure, and diabetic nephropathy [11,12]. Despite its efficacy, CAP suffers from low and variable oral bioavailability (60–75%), mainly due to extensive hepatic metabolism and reduced absorption in the presence of food [13]. However, the incorporation of captopril into an oral film formulation may offer a great challenge because some authors reported that captopril falls into BCS class III where poor permeability becomes a rate-limiting step in the absorption process [14]. These characteristics, in addition to its low molecular weight [15], make CAP a relevant model drug for investigating strategies aimed at improving epithelial transport using buccal film systems.

Chitosan (CHI), a biodegradable and biocompatible polysaccharide derived from chitin, has been extensively explored in the mucoadhesive drug delivery carrier due to its unique cationic nature [16]. Its ability to form ionic interactions with negatively charged sialic acid residues in mucin enhances its mucoadhesiveness [17]. Additionally, CHI has been reported to improve drug permeability across mucosal tissues by transiently opening tight junctions, making it a valuable excipient in transmucosal formulations [18,19].

However, CHI is soluble only in acidic media, which may pose formulation challenges. To address this, weak organic acids, such as ascorbic acid (AA), can be used to solubilize CHI and modulate its physicochemical behavior by the formation of chitosan–ascorbate (CHI-Asc) complexes via ionic interactions of this dosage-form system [20,21]. Previous studies have reported that CHI-Asc systems may enhance epithelial drug transport compared with other chitosan salts. One possible explanation for this behavior is that AA enhances the ability of CHI to interact with extracellular lipids, which constitute the main barrier to drug transport across the buccal mucosa via paracellular route [22]. Additionally, AA possesses antioxidant activity [23], which may protect sensitive active ingredients such as CAP from oxidative degradation during processing and storage. However, the overall stability of the dosage form remains dependent on environmental conditions.

Glycerol (GLY) is widely used as a plasticizer in film-based drug delivery systems. It improves flexibility and decreases fragility of polymeric films by reducing intermolecular hydrogen bonding, and has shown good compatibility with CHI-based matrices [24].

In our previous work, mucoadhesive buccal films composed of CHI (1.4%), AA (2.5%), and GLY (30% *w*/*w* of CHI) were formulated and characterized, incorporating CAP at therapeutic doses of 10 mg and 20 mg per 4 cm^2^ film unit [21]. These films demonstrated uniform drug distribution, immediate-release kinetics, and acceptable mucoadhesive and mechanical performance based on quality by design (QbD) and design of experiments (DoE) approaches.

Although initial formulation efforts confirmed the feasibility of this system, further investigation is required to determine its biocompatibility, permeability, and stability under conditions that simulate the real application. In this context, cytotoxicity testing is a critical step in the development of mucosal delivery systems, ensuring that the formulation does not compromise cellular viability [25]. Moreover, permeability testing using buccal epithelial cell lines (e.g., TR146) is essential to predict drug-absorption behavior [26]. Finally, the evaluation of accelerated stability provides information on the physical and chemical integrity of the films over time [27].

While the CHI-Asc system has previously been investigated in solution form for permeation and cytotoxicity studies [22], its translation into a buccal-film dosage form introduces formulation-specific considerations that cannot be inferred from solution-based experiments alone. Therefore, the novelty of the present work lies not in the polymer chemistry itself, but in the integrated in vitro assessment of cytocompatibility, permeability, and accelerated stability of CAP-loaded CHI-Asc buccal films, providing a more comprehensive characterization of this formulation approach. The results of cytocompatibility and permeability were compared with those of Chitosan–acetate (CHI-Ac) films, which were selected as a reference system because acetate is one of the most commonly used counter ions for chitosan in buccal and mucosal delivery systems [28,29,30,31]. Using CHI-Ac as a comparator allows assessment of the specific influence of the ascorbate counter ion on film cytotoxicity and epithelial transport while maintaining the same polymer backbone.

Therefore, the present study was carried out to evaluate the cytotoxicity, permeability and stability of the previous developed CHI-Asc-based buccal films loaded with CAP in order to further define the performance and limitations of this dosage-form system.

## 2. Results and Discussion

### 2.1. Cytotoxicity Test

**Figure 1:**
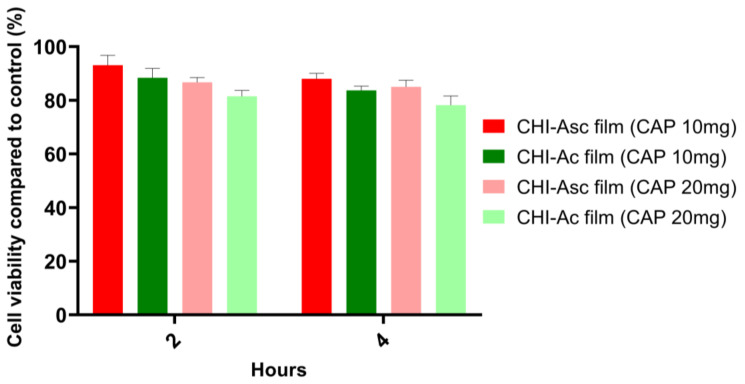
Cytotoxicity of the prepared films measured by the NR assay. Cell viability expressed as the percentage of absorbance of the untreated control cells. Data expressed as mean ± SD, *n* = 12.

TR146 cells were used as an in vitro model to mimic the stratified epithelium of human buccal mucosa [32,33]. TR146 cells were treated with the films added on top of 100 µL HBSS buffer solution for 2 and 4 h and the toxicity study was performed using the NR assay. In this assay, viable cells retain NR dye in lysosomes, while compromised cells exhibit poor or no capacity to pick up NR due to loss of membrane integrity or lysosomal function [34]. Figure 1 shows the results of the cytotoxicity test according to the NR uptake method. It can be seen that all formulations have viability values greater than 78% compared to untreated control cells. There was no statistically significant difference between the results. On the basis of our results, these formulations could be considered safe and suitable for buccal application [35].

These findings are in line with previous reports on the biocompatibility of CHI derivatives. CHI-Ac cytotoxicity was evaluated using TR146 buccal cell lines in the short term (4 h) and showed high cell viability, indicating non-toxicity [36]. CHI-Asc also exhibited low cytotoxicity in Caco-2 intestinal cells, with cell viability percentages comparable to those of chitosan hydrochloride and lactate, which are known as salts characterized by low toxicity [22]. Furthermore, viability values remained consistent over different exposure durations, supporting the safety of CHI-Asc for pharmaceutical applications [22].

### 2.2. In Vitro Permeation Test

TR146 cells have been widely utilized as an in vitro model for studying buccal drug permeability and transport mechanisms [37], including studies evaluating the impact of permeation enhancers [38].

**Figure 2:**
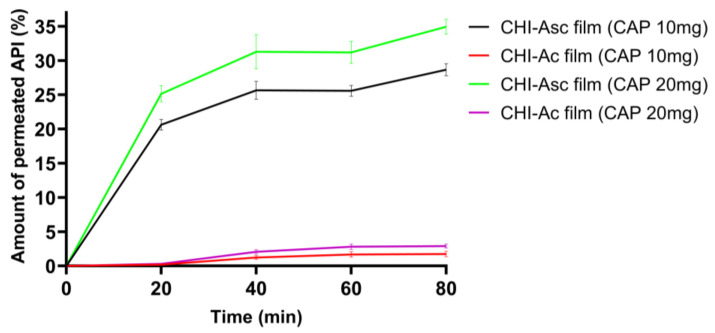
CAP permeated the amount–time curves of the buccal films on the TR-146 cell line. Each film was investigated in three separate, parallel cell culture inserts.

In the present work, the feasibility of CAP permeation across the buccal epithelium from CHI-Asc films was investigated using the TR146 cell culture model and compared with that from CHI-Ac films. The cumulative permeation profiles of CAP from all film formulations across TR146 cells are presented in Figure 2.

As can be seen in Figure 2, the permeation of CAP from CHI-Asc films was markedly higher than that from CHI-Ac films at both drug loadings. For the ascorbic acid-containing films, a rapid burst of API could be detected within the first 20 min, followed by a slower and sustained permeation phase, reaching approximately 32–34% of the applied dose after 80 min. In Table 1. flux and permeability values were calculated for each sampling time and the steady-state period of 20–80 min as well. The 10 mg CAP CHI-Asc films exhibited a similar trend but with a lower cumulative permeation, reaching approximately 25–28% during the same period. On the contrary, the CHI-Ac films showed minimal transport of CAP, with cumulative permeation remaining below 2–3% regardless of drug load. An increase in CAP loading from 10 mg to 20 mg resulted in higher cumulative permeation for both polymer systems, particularly for CHI-Asc films. This dose-dependent behavior is consistent with passive diffusion-driven transport, where an increased concentration gradient across the epithelial barrier enhances drug flux under sink conditions. This behavior is considered the primary mechanism for drug absorption from the oral cavity, and only a limited number of cases of active transport have been reported, which involved a nutrient with stereospecific properties [39]. Average flux and apparent permeability values were at least tenfold higher in the case of ascorbic acid-containing films, than in the case of acetic acid-containing ones.

These results demonstrate that drug concentration and polymer salt form influence CAP transport across the TR146 buccal epithelial model. The difference in the transported CAP amount indicates that the ascorbate salt form of CHI is capable of improving CAP transport in the in vitro experiment, which may be associated with modulation of epithelial barrier properties, although the specific transport mechanism was not directly investigated in this study. On the contrary, the very low permeated API quantity, flux and permeability observed from CHI-Ac films suggests that this salt form is less effective as a permeation enhancer for CAP under the applied conditions and it also limits the release of CAP from the matrix.

This interpretation is further supported by comparison with the published literature. A study investigating the buccal permeation of pramipexole from CHI-Ac-based films across TR146 cells reported cumulative permeation values that are comparable to those obtained in the present work for CAP from CHI-Asc films [36]. Importantly, pramipexole is classified as a BCS class I drug [40], characterized by high intrinsic permeability. The observation that captopril exhibited permeation levels similar to those of pramipexole only when formulated with CHI-Asc strongly supports the conclusion that CHI-Asc significantly enhances CAP permeability, rather than the drug being intrinsically permeable. Therefore, the present results collectively indicate both the low inherent buccal permeability of CAP and the superior permeation-enhancing property of CHI-Asc compared to CHI-Ac in the TR146 buccal epithelial model.

The initial and the latter phases of release of API can be explained by the liberation from the formulated films. The films are placed on the surface of the apical chamber of the cell culture insert, filled with buffer solution. There is a quick, burst release of CAP from the ascorbic acid films, while there is only a very limited release from the acetic acid films, confirming the enhanced liberation of CAP from the CHI-Asc samples. Yet, following this period, a much slower release can be monitored from 20 to 80 min, where the main limiting factor is no longer the release of the API from the matrix, but the saturated solution of CAP in the buffer media permeating through the cell layer. The average flux and apparent permeability values shown in Table 1 prove that during its biphasic permeation profile, CHI-Asc samples have high flux, permeability and permeated API values, showing that ascorbic acid not only promotes the release of the API from the matrix, but also acts as a permeation enhancer. Acetic acid failed to achieve a saturated solution in the apical chamber, due to the very limited release of the API from the respective matrix. It must be noted that the static donor phase without stirring leads to a non-saturated solution which lowers permeated API amount and does not mimic in vivo conditions where the constant washing effect of saliva is present, which could have increased permeation of the API from CHI-Ac samples [41].

As both films utilize chitosan as the carrier polymer, the difference in permeation lies in the interaction of acetic acid and ascorbic acid with chitosan. The unique positive interaction of chitosan and ascorbic acid is further supported by the results of Rossi et al., who observed improved penetration enhancement properties of chitosan ascorbate compared to lactate and hydrochloride salts on Caco-2 cells using fluorescein isothiocyanate-dextran as a model API [22]. Further research proved the enhanced effect of chitosan ascorbate nanoparticles for the vaginal delivery of amoxicillin [42]. Two studies confirmed the enhancement effect of chitosan on epithelial permeability through the modulation of tight-junction integrity. Chitosan induced a reversible decrease in transepithelial electrical resistance, accompanied by enhanced paracellular transport of the paracellular markers, indicating disruption of tight junctions and confirming its effect on the paracellular pathway. These changes were associated with structural changes at the cellular level, including reorganization of the actin cytoskeleton and alterations in tight-junction proteins (ZO-1 and occludin), indicating a transient opening of the epithelial barrier [43,44]. This translocation of tight-junction proteins (ZO-1 and occludin) from the plasma membrane into the cytoskeletal fraction indicates a loss of their functional role in maintaining barrier integrity, and suggests a direct interaction of chitosan with cellular structures, which provides evidence that chitosan enhances permeability primarily through structural disruption of tight-junction complexes at the molecular level [45]. Another microscopic and ultrastructural experimental study also demonstrated that chitosan enhanced epithelial permeability by translocating and disrupting tight-junction proteins (JAM-1), accompanied by a significant decrease in transepithelial electrical resistance, indicating increased paracellular permeability. Importantly, these effects were found to be transient and reversible upon removal of chitosan, with full recovery of tight-junction integrity. In vivo studies additionally revealed that while chitosan remains primarily associated with the epithelial surface due to its mucoadhesive properties, it facilitates the transport of coadministered molecules through the paracellular pathway, supporting its role as a safe and effective permeation enhancer [46].

Collectively, these findings highlight that the presence of ascorbic acid synergistically improves the effects of chitosan as an effective permeability enhancer through modulation of epithelial tight junctions. Considering the physicochemical properties of captopril as a hydrophilic drug with limited membrane permeability, its transport is expected to rely on the paracellular route [47]. Therefore, the enhanced permeability observed in our study may be explained by the ability of the formulation to facilitate paracellular transport, consistent with previously reported mechanisms of chitosan. Meanwhile, the release of CAP was severely limited from the CHI-Ac samples, which resulted in the low permeated API amount.

### 2.3. Stability Results

The accelerated stability test was performed at 40 °C and 75% relative humidity (RH) for three months to evaluate the effect of environmental stress on the mechanical and physicochemical stability of the prepared films. This approach enables early detection of changes in drug content, moisture uptake, film thickness, mechanical strength, and mucoadhesive performance, which are critical quality attributes for buccal delivery systems. Monitoring these parameters over time provides insight into the intrinsic formulation sensitivity to elevated temperature and humidity. It should be noted that this study was designed as an exploratory accelerated stress evaluation rather than a formal ICH-compliant packaging simulation.

#### 2.3.1. Moisture Content and Film Thickness

Changes in moisture content and film thickness are shown in Table 2. Moisture absorption increased progressively with storage, and blank films showed the highest increase (from 3.3% to 14.7% at 3 months). In the case of 60- and 90-day samples, the moisture content increased significantly, and in the case of the 10 mg sample, even after 30 days. This reflects the known hygroscopic nature of CHI and AA, which readily absorb atmospheric moisture at elevated humidity [48,49]. The increase in water content led to swelling of the polymer matrix, explaining the observed increase in film thickness, particularly in drug-loaded samples (up to 230 µm). Such hydration-induced swelling and relaxation have previously been reported in CHI films and are associated with reduced matrix rigidity [50].

#### 2.3.2. Mechanical Properties (Breaking Hardness, Puncture-Resistance Time, and In Vitro Mucoadhesivity Test)

Fresh films exhibited adequate breaking hardness, puncture-resistance time, and in vitro mucoadhesive force. However, over storage, there was a decrease in these properties (Table 3). The breaking hardness decreased for 10 mg CAP films from 18.8 N to 6.0 N after 30 days and 5.0 N after 90 days, and from 14.4 N to 5.2 N after 30 days and 3.2 N after 90 days in the 20 mg CAP films. The puncture-resistance time was also reduced over this period. The puncture-resistance time of the 10 mg CAP films changed from 11.5 s to 8.0 s after 30 days and to 4.0 s after 90 days, and from 12.0 s to 8.7 s after 30 days and to 4.3 s after 90 days in the case of the 20 mg CAP films. These results support the loss of flexibility and increased brittleness of the films, which is a normal behavior of aging polymers [51]. The mucoadhesion force also decreased in all cases. In the case of the 10 mg CAP films, freshly prepared films showed an in vitro mucoadhesion force of 15.1 N which changed to 5.8 N after 90 days. In the case of the 20 mg CAP films, the in vitro mucoadhesion force decreased from 18.5 N, which was for freshly prepared films, to 8.2 N after 90 days. This phenomenon can probably be explained by the fact that there are fewer free functional groups available during storage as a result of the high water uptake and new interactions, so carboxyl and hydroxyl groups cannot connect to the mucin chain of the buccal mucosa. This observation is also supported by the results of the breaking hardness of the films because breaking hardness of all films decreased during storage, so that the cohesive structure could be changed by the forced condition.

The breaking hardness, puncture resistance and mucoadhesive force decreased significantly (*p* < 0.05) during storage in all cases except for the blank film, where there was no significant decrease in breaking hardness after 30 days. The reduction in mechanical strength and puncture-resistance time during storage is likely associated with increased moisture uptake, which acts as a plasticizing agent within the chitosan matrix. Water molecules can disrupt intermolecular hydrogen bonding between polymer chains, leading to reduced structural cohesion and increased brittleness. Similar behavior has been reported for chitosan-based films under humid conditions [52,53].

Such mechanical weakening during accelerated storage was reported in CHI films and tablets stored at high humidity. Viljoen et al. found that the mechanical properties of CHI powder and tablets deteriorated significantly, especially under accelerated conditions of high temperature and humidity (40 °C/75% RH) due to dehydration and aging of CHI particles [54]. Cervera et al. found that the CHI-amylose starch films stored under these conditions absorbed more moisture, making the films mechanically weaker [55]. Furthermore, excessive moisture at high relative humidity could also reduce the mucoadhesive strength of CHI-based carriers as a result of the ‘dilution’ of functional groups (such as hydroxyl or amino groups) needed to interact with mucin. In other words, these groups become less concentrated or less accessible for bonding due to the excessive presence of water [56].

In general, under accelerated storage conditions, the mechanical properties of the CAP-containing buccal films deteriorated progressively over time; therefore, in selecting the most suitable storage conditions, the use of proper airtight packaging should also be considered to protect hygroscopic chitosan products from environmental humidity.

#### 2.3.3. Drug Content

**Figure 3:**
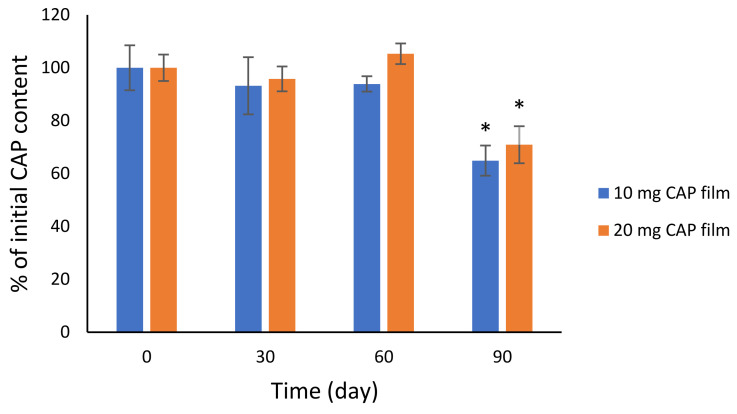
CAP content, expressed as a percentage of the initial value (day 0 = 100%), in CHI-Asc films during the accelerated stability period. Data expressed as mean ± SD, *n* = 3 (* *p* < 0.05).

CAP contains a thiol (–SH) group, making it highly prone to oxidative degradation, particularly in aqueous or high-moisture environments [57,58]. The results of the drug assay demonstrated that CAP content decreased to 64.9 ± 5.7% and 70.9 ± 7.0% of the initial content for the 10 mg and 20 mg films, respectively after 90 days of storage under accelerated conditions, as shown in Figure 3. The results showed a significant difference (*p* < 0.05) only after 90 days. This degradation correlates with the observed increase in the moisture content of the film (10 mg film: from 10% to 12.7%; 20 mg film: from 3.5% to 11.9%), suggesting that elevated moisture levels, in addition to relatively high temperature (40 °C)—accelerate oxidative pathways indirectly, can lead to degradation of captopril. Several scientific articles mention and demonstrate the oxidative degradation pathway in aqueous solution [57,58].

However, the CAP content decreased only to a small extent after 60 days of storage under accelerated conditions (to 93.9 ± 2.9% and 105.3 ±3.9% of the initial content for the 10 mg and 20 mg films, respectively), as can be seen in Figure 3. The slightly higher drug content measured on day 60 compared to the initial value does not indicate an increase in drug quantity; however, on day 90 the difference is already significant (*p* < 0.05) (Figure 3). Drug content was determined on independently sampled film units at each time point, and the observed difference falls within acceptable pharmacopeial content uniformity limits (85–115%). To conclude, these results showed that each film retains almost all amount of API content (˃90%) for 60 days without any decomposition, which meets the suitable extent of content according to the European Pharmacopeia (Ph.Eur.) [59].

The observed decrease in CAP content in CHI-Asc buccal films during accelerated storage conditions (elevated temperature and relative humidity) showed a clear positive correlation with increased moisture levels in the films. This suggests that water uptake enhanced molecular mobility within the polymer matrix, thereby facilitating degradation processes of the embedded API.

Chitosan and its salt derivatives, such as CHI-Asc, are inherently hydrophilic due to the abundance of hydroxyl and amino groups capable of forming hydrogen bonds with water molecules. This hygroscopic nature leads to significant moisture sorption, especially at high relative humidity, which plasticizes the polymer chains. Plasticization increases free volume and promotes segmental mobility, allowing greater diffusion of oxygen, water, and drug molecules within the matrix. Consequently, degradation reactions of embedded drugs are accelerated [60].

Similar observations appear in studies of captopril formulations. In HPMC-based orally disintegrating films, captopril stability was noted to depend strongly on pH (<4 for optimal stability), temperature, humidity, and the presence of hygroscopic excipients, with oxidative degradation to the disulfide being the dominant route. Low water activity and protective packaging were recommended to minimize degradation [61]. In mucoadhesive buccal patches and other polymeric systems, excessive moisture uptake has been linked to potential hydrolytic degradation of captopril, underscoring the need for controlled humidity during storage [62].

In the context of the present CHI-Asc films, the ascorbate component may provide some antioxidant protection; however, at elevated humidity, the overall plasticizing and mobilizing effect of water apparently outweighed this benefit, resulting in net loss of CAP content. These findings emphasize the importance of optimizing film composition (e.g., through cross-linking, hydrophobic additives, or desiccants in packaging) and selecting appropriate storage conditions (preferably low temperature and low RH) to ensure long-term stability of moisture-sensitive drugs in chitosan-based buccal delivery systems.

However, the amount of CAP decreased only slightly after 60 days of storage under accelerated conditions (to 93.9 ± 2.9% and 105.3 ± 3.9% of the initial content for the 10 mg and 20 mg films, respectively), as shown in Figure 3. This limited degradation correlated well with the relatively modest increase in moisture content observed during the same period (from initial values of 3.3–4.0% to 5.8–7.7% across blank and CAP-loaded films; see Table 2).

#### 2.3.4. FT-IR Spectroscopy

**Figure 4:**
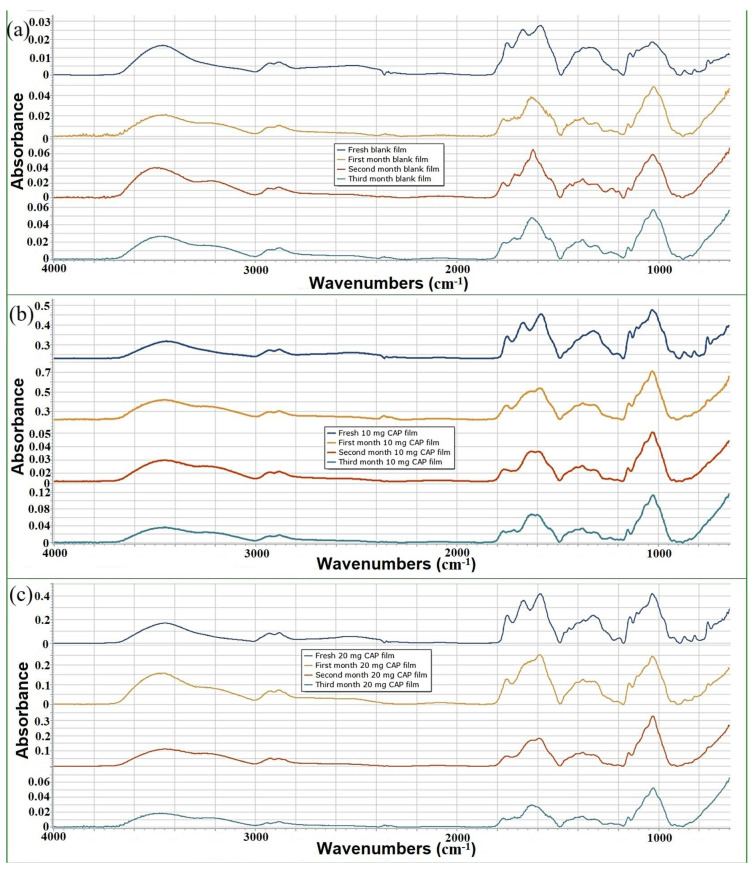
FT-IR spectra of fresh and stored (0–3 months at 40 °C, 75% RH) buccal films: (**a**) blank film, (**b**) 10 mg CAP film, (**c**) 20 mg CAP film.

FT-IR spectra of the blank and CAP-loaded films were recorded for fresh films and after 30, 60 and 90 days at 40 °C/75% RH (Figure 4). FT-IR analysis of fresh films showed the expected band at ≈3600–3200 cm^−1^ which is related to the stretching vibration of the -OH groups of AA, GLY, and CHI and to the N-H stretching vibration of CHI [21]. The sharp –SH characteristic peak of CAP, which is 2567 cm^−1^, cannot be observed in the polymer film system due to interactions created between the other excipients of the films in the form of hydrogen bonds [63]. However, the presence of this peak was demonstrated in polymer films by RAMAN spectroscopy in our previous work and the presence of CAP was also demonstrated via drug content tests (Figure 3) so that CAP can be found in the polymer film system without any decomposition [21].

During accelerated storage, the analysis demonstrated that all spectra, regardless of the content of CAP, exhibited the characteristic stretching band of O–H/N–H (3600–3200 cm^−1^) that progressively intensified during storage, indicating increased moisture uptake (consistent with moisture uptake, Table 2) and stronger hydrogen bonds can be created within the polymeric matrix. In blank films, slight peak shifts to higher wavenumbers were observed in the 1750–1600 cm^−1^ region, whereas peak overlap occurred in CAP-loaded films between the chitosan C=O of amide I at ~1650 cm^−1^ and the N–H of amide II at ~1585 cm^−1^ (Figure 4) [64]. These changes are consistent with an increase in hydration and a hydrogen-bond rearrangement of the chitosan matrix. However, no significant differences or new intense IR bands indicative of major new chemical reactions or interactions were observed, suggesting that the polymer system did not undergo degradation and decomposition. In conclusion, FT-IR data indicate that the films absorbed moisture under the accelerated storage conditions and formed new hydrogen bonds, yet remained structurally stable.

## 3. Materials and Methods

### 3.1. Materials

Medium molecular weight CHI (average MW: 1250 kDa; batch number: 889EMW) (≥90% degree of deacetylation) was used as film-forming polymers and was purchased from Glentham Life Sciences Ltd. (Corsham, UK). GLY (Ph. Eur.9.0.; ≥99.5%), added to the film as a plasticizer, was supplied by Merck (Darmstadt, Germany). AA (Ph.Eur.9.0; Lot No. D19408696), added to the film to dissolve CHI and enhance permeation, was purchased from Molar Chemicals Ltd. (Halásztelek, Hungary). CAP (Ph.Eur.9.0; Lot No. 460500723) was obtained as a gift from EGIS Pharmaceuticals PLC (Budapest, Hungary). Acetic acid (min. 99.8%, Sigma-Aldrich, Darmstadt, Germany) provided an appropriate acidic media for CHI dissolution during the preparation of the reference films. Mucin Type II porcine stomach mucin, (10 *w*/*w*%) dispersion used in the in vitro mucoadhesion test, was purchased from Sigma-Aldrich (St. Louis, MO, USA). Distilled water was used as a solvent in the preparation of the solutions.

### 3.2. Preparation of Buccal Films

Films were prepared employing the solvent casting method. Table 4 shows the composition of the prepared polymer film solutions. The required amount of CHI was dispersed in the acidic aqueous solution and mixed (Stirrer DLS, VELP Scientifica, Usmate, Italy) at 900 rpm using a propeller-type mixing rod until complete dispersion. Then the solution was left for about four hours to completely dissolve. After a clear solution was formed, the required amount of CAP was added and mixed. The calculated amount of plasticizer, based on the dry weight of the polymer, was then added to the solution and stirred for 20 min. A predetermined amount of the solution was poured onto plastic Petri dishes (diameter: 9 cm) and allowed to dry in a ventilated oven (Memmert GmbH + Co. KG, Schwabach, Germany) under predetermined conditions (temperature: 30 °C, airflow: 30% fan capacity). A part of the dried films was placed in the incubator for stability testing and the other part was carefully sealed until further investigation. CHI-Ac films were prepared under comparable formulation conditions and used as a reference in in vitro permeation and cytotoxicity tests.

### 3.3. Permeation and Cytotoxicity Test

#### 3.3.1. Cell Culture

TR146 cells (European Collection of Authenticated Cell Culture Catalogue No: 10032305) were cultured in Dulbecco’s modified Eagle’s medium with L-glutamine and D-glucose supplemented with 10% (*v*/*v*) FBS, 3.7 g/L sodium hydrogen carbonate, 1% (*v*/*v*) non-essential amino acid solution and 100 IU/mL penicillin K, with 100 μg/mL streptomycin sulfate at 37 °C in an atmosphere of 5% CO_2_ in plastic cell culture flasks. Cells were routinely maintained by regular passaging from passage number 22 to 32.

#### 3.3.2. Cytotoxicity Test

The cytotoxicity of all samples was assessed with Neutral Red (NR) Uptake assay. Cells were seeded in 96-well plates at a final density of 10.000 cells/well and after 7 days, they were incubated with films cut to 7.06 mm^2^ area films for 2 and 4 h respectively with 100 µL HBSS. After film removal, cells were washed with phosphate-buffered saline and 100 µL of 33.3 mg/mL NR solution (NR obtained from Alfa Aesar (Karlsruhe, Germany) solved in cell culture medium) was added to each well. Cells were incubated for 2 h, then the NR solution was removed and 0.1 mL of a solution of isopropanol—1 M hydrochloride acid (25:1) was added to each well to dissolve cells. Empty wells of the plate were used as a background and all measurements were carried out with a Thermo-Fisher Multiskan Go microplate reader (Thermo-Fisher, Waltham, MA, USA) at a wavelength of 540 nm. Cytotoxicity values were compared to cells treated with cell culture media.

#### 3.3.3. In Vitro Permeation Test

Permeation test started as 1 × 10^5^ cells were seeded on ThinCert^®^ PET cell culture inserts with 0.4 µm pore size diameter, 2 × 10^6^/cm^2^ pore density on a 33.6 mm^2^ culturing surface (Greiner BioOne, Mosonmagyaróvár, Hungary). During the two weeks of maturation, fresh culture medium was added to the cells every 2 days. A Millicell ERS 1 device was used to measure transepithelial electrical resistance regularly (Merck, Budapest, Hungary). Only inserts with a resistance value over 130 Ω*cm^2^ were used for the permeation tests. The films were cut to an equal weight with a size of 7.06 mm^2^ and their CAP content was 0.1765 mg for 10 mg CAP films and 0.3530 mg for 20 mg CAP films. First, the cell culture medium was removed and 400 µL of Hank’s balanced salt solution (HBSS) was added to the apical compartment (AC) and 1000 µL of HBSS to the basolateral compartment (BC) of each selected insert. The experiment started when a film was added to the top of the AC. 200 µL of HBSS was removed from the BC every 20 min and the removed volume was replaced with fresh solvent. The samples were introduced into the LC/MS chromatographic system consisting of a Waters 2695 Separations Module with a thermostable auto sampler (5 °C) and a column module (35 °C), a VDSphere PUR 100 C18-M-SE reverse phase C-18 column (4.6 × 150 mm, 5 μm) (VDS Optilab Chromatographie Technik GmbH, Berlin, Germany) coupled with a MicroTOF-Q type Qq-TOF MS instrument (Bruker Daltonik, Bremen, Germany) using an electrospray ion source (ESI) with positive ion mode. The flow rate and the injected volume were 1.0 mL/min and 10 µL, respectively, in all cases. A splitter was applied between the HPLC and MS, obtaining a flow rate of 0.1 mL/min for the ESI ion source. The mass spectra were calibrated externally using the exact masses of the clusters generated from the electrosprayed solution of sodium trifluoroacetate (NaTFA). Eluent A was 0.1% trifluoroacetic acid (TFA) in water, eluent B was acetonitrile. The eluent table was the following: 0 min and 5 min 80% A, 20% B; 15 min and 20 min 30% A, 70% B; 20 min and 25 min 80% A, 20% B. The recorded mass spectra were evaluated with Bruker Data Analyzer 3.4 software.

Average flux values for the transport were calculated as:

(1) $$\frac{Q_{n}}{{A\;\times \Delta t}_{a}}=J$$

where *Q_n_* is the cumulative mass of transported API calculated for each sampling time in mg (considering the previously taken sample amounts for 40, 60 and 80 min), *A* is the surface area of the cell culture insert in cm^2^, Δ*t* is the time between sampling in minutes.

Apparent permeability flux values for the transport were calculated as:

(2) $$\frac{\Delta Q}{{A\;\times \Delta t\times C}_{0}}=P_{app}$$

where Δ*Q* is the cumulative amount of transported API calculated for each sampling time in mol (considering the previously taken sample amounts for 40, 60 and 80 min), *A* is the surface area of the cell culture insert in cm^2^, Δ*t* is the time between sampling in seconds, *C***_0_** is the initial concentration of the API in the apical compartment in mol.

### 3.4. Stability Test

An accelerated stability test for the CHI-Asc films was performed, and at each stability time point, three independent film units were randomly selected and analyzed. Films were kept in closed Petri dishes and stored in a humidity chamber (75 ± 5% RH), which was placed in an incubator (Heraeus Hanau B-6 Function Line Laboratory Incubator, Heraeus Hanau, Hanau, Germany), maintained at 40 ± 2 °C for a period of 3 months [65]. This setup was designed to evaluate moisture sensitivity and mechanical stability under controlled environmental exposure rather than to simulate final pharmaceutical packaging in accordance with ICH stability guidelines. Changes in thickness, moisture content, breaking hardness, puncture-resistance time, in vitro mucoadhesivity, and drug content were analyzed at a regular interval for 3 months. Furthermore, the interactions were also examined with FT-IR spectroscopy during the three-month period.

#### 3.4.1. Moisture Content and Film Thickness

The moisture content of the films was determined using a halogen moisture analyzer (MAC 50/NH, RADWAG, Radom, Poland). Three parallel measurements were performed for each film using a drying temperature of 105 °C and automatic switch off mode 3. The results were expressed in percentage.

The thickness of each film was measured using a micrometer screw gauge with a measurement range of 0 to 25 mm and a resolution of 0.001 mm (Mitutoyo Co., Ltd., Kanagawa, Japan) at five different points, and the average values and standard deviations were calculated.

#### 3.4.2. Breaking Hardness

The breaking hardness of the films was measured using a texture analyzer developed at our institute [66]. This equipment is equipped with various sample holders and probes tailored for different types of tests. To measure the breaking hardness of the film, the sample was placed in the holder at the base of the equipment, and a needle-like probe (3 mm in diameter) was moved downward at a constant speed (20 mm/min) against the film. The needle was stopped when the film broke, recording both the duration of the test and the force required to break the film (ranging from 0 to 50 N). The results were displayed as a force–time curve on a computer connected to the equipment. The test was repeated three times for each formulation, with mean values and standard deviations calculated [67].

#### 3.4.3. Puncture-Resistance Time

The puncture-resistance time was determined from the force–time curve of breaking hardness. The duration from the onset of needle penetration to the completion of the film puncture was taken as an indirect measure of flexibility and was recorded in seconds [68]. Each formulation was tested three times, and the results are reported as mean values with the corresponding standard deviations.

#### 3.4.4. In Vitro Mucoadhesivity of Films

The in vitro mucoadhesion test was performed using the same texture analyzer with different accessories and parameters. A rod-like probe (9 mm in diameter) was used as a sample holder, and a double-faced adhesive tape was used to fix the film on the bottom side of this probe. Then 25 µL of a freshly prepared mucin solution (10% *w*/*w*) was spread on a disk (11 mm in diameter) assembled at the bottom of the equipment. The rod-like probe was moved downwards on the disk, and a force of 30 ± 0.1 N was applied for 30 s to ensure the film made close contact with the mucin. The probe was then moved upward, and the mucoadhesive force was recorded as a peak in the force–time curve. The test was repeated three times for each formulation and means and standard deviations were calculated [69].

#### 3.4.5. Drug Content

The drug-containing films were prepared by loading an adequate amount of the drug to be the final amount of captopril of 10 mg and 20 mg per each 4 cm^2^ of the film. To assess the drug content of CAP, films of 2 × 2 cm^2^ were dissolved in 100 mL of phosphate buffer (PB) (pH 6.8) and stirred for 1 h. The CAP concentration was then determined with a Genesys 10S UV-VIS spectrophotometer (Thermo Fisher Scientific, Waltham, MA, USA) at 208 nm.

#### 3.4.6. FT-IR Spectroscopy

Fourier transform infrared (FT-IR) spectroscopy was used to give insight into chemical changes and interactions in the film formulation during the stability test period. An Avatar 330 FT-IR apparatus (Thermo Fisher Scientific Inc., Waltham, MA, USA) coupled with a Zn/Se horizontal attenuated total reflectance (HATR) accessory was used. The samples, placed on the crystal of the equipment, were scanned for absorbance in the wavelength range of 600 cm^−1^ to 4000 cm^−1^ [69]. The spectra were obtained by applying H_2_O and CO_2_ corrections with a spectral resolution of 32 cm^−1^ from 128 scans. To evaluate the results, SpectraGryph (version 1.2.15.; Dr. Friedrich Menges Software, Entwicklung, Obersdorf, Germany) was utilized.

### 3.5. Statistical Analysis

The significance tests for breaking hardness and in vitro mucoadhesivity were performed with Microsoft Excel (version 15, Redmond, Washington, DC, USA) software. A Two-Sample *T*-Test was applied. In all cases, the samples were compared to the freshly prepared sample. Calculations were carried out by GraphPad Prism software (version 8, GraphPad Software, San Diego, CA, USA). In all cases, we analyzed data sets with the Kruskal–Wallis test with Dunn’s test as post hoc test. In each case, we used a significance level of *p* < 0.05. Significance is labeled as ns = *p* ≥ 0.05; * = *p* < 0.05 ** = *p* < 0.01.

## 4. Conclusions

In this work, previously developed CHI-Asc buccal films loaded with CAP were evaluated with respect to cytotoxicity, enhancement of CAP permeability, and stability. The in vitro cytotoxicity results confirmed that the formulated films are well tolerated by TR146 cells, exhibiting cell viability of over 80% in the case of all samples, highlighting the low toxicity and a good safety profile. The permeability test demonstrated that the CHI-Asc films significantly enhanced the buccal permeation of the CAP compared to the CHI-Ac films by up to a 12–16-fold value. Stability investigations under accelerated storage conditions revealed that the films undergo progressive moisture uptake over time, leading to changes in thickness, mechanical properties, mucoadhesive force, and a decrease in CAP content after 3 months, but they were able to retain almost all of their active ingredient content (˃90%) after 60 days. However, FT-IR spectroscopy indicated that the observed changes were mainly physical and moisture-induced, with no significant decomposition or structure changes.

Overall, the findings of this work provide an in vitro, dosage-form–level characterization of CHI-Asc buccal films, and highlight their cytocompatibility, and permeability-enhancing potential.

## Figures and Tables

**Table 1:** Average flux (**J** in mg/min × cm^2^) and apparent permeability (**P_app_** in cm/s) values of CHI-Asc Film (CAP 20 mg) and CHI-Ac Film (CAP 20 mg) formulations.

Sample No.	20 min	40 min	60 min	80 min	20–80 min
**J** of CHI-Asc Film (CAP 10 mg)	6.293 × 10^−3^	7.199 × 10^−3^	9.094 × 10^−3^	1.081 × 10^−2^	9.032 × 10^−3^
**J** of CHI-Ac Film (CAP 10 mg)	8.095 × 10^−5^	5.121 × 10^−4^	7.642 × 10^−4^	8.542 × 10^−4^	7.102 × 10^−4^
**P_app_** of CHI-Asc Film (CAP 10 mg)	2.971 × 10^−4^	3.399 × 10^−4^	4.291 × 10^−4^	5.103 × 10^−4^	1.280 × 10^−3^
**P_app_** of CHI-Ac Film (CAP 10 mg)	3.822 × 10^−6^	2.418 × 10^−5^	3.608 × 10^−5^	4.033 × 10^−5^	1.006 × 10^−4^
**J** of CHI-Asc Film (CAP 20 mg)	1.321 × 10^−2^	1.512 × 10^−2^	1.909 × 10^−2^	2.270 × 10^−2^	1.897 × 10^−2^
**J** of CHI-Ac Film (CAP 20 mg)	1.700 × 10^−4^	1.075 × 10^−3^	1.605 × 10^−3^	1.794 × 10^−3^	1.491 × 10^−3^
**P_app_** of CHI-Asc Film (CAP 20 mg)	6.239 × 10^−4^	7.138 × 10^−4^	9.012 × 10^−4^	1.072 × 10^−3^	2.687 × 10^−3^
**P_app_** of CHI-Ac Film (CAP 20 mg)	8.026 × 10^−6^	5.077 × 10^−5^	7.578 × 10^−5^	8.470 × 10^−5^	2.112 × 10^−4^

**Table 2:** Moisture content and film thickness of CHI-Asc films during the accelerated stability period (data expressed as mean ± SD, *n* = 3 for moisture content and *n* = 5 for thickness) (* *p* < 0.05).

Time (Day)	Moisture Content (%)			Film Thickness (µm)		
	Blank Film	10 mg CAP Film	20 mg CAP Film	Blank Film	10 mg CAP Film	20 mg CAP Film
0	3.3 ± 0.6	4 ± 0.4	3.5 ± 0.6	143 ± 23	157 ± 14	192 ± 22
30	4.8 ± 0.3	6.4 ± 0.9 *	5.1 ± 0.8	131 ± 29	159 ± 46	213 ± 43
60	5.8 ± 0.9 *	7.7 ± 1.1 *	6.1 ± 0.8 *	139 ± 28	200 ± 59	223 ± 63
90	14.7 ± 1 *	12.7 ± 0.3 *	11.9 ± 0.9 *	143 ± 36	196 ± 50	230 ± 41

**Table 3:** Breaking hardness, puncture-resistance time, and mucoadhesion force of CHI-Asc films during the accelerated stability period (Data expressed as mean ± SD, *n* = 3) (* *p* < 0.05).

Time (Day)	Breaking Hardness (N)			Puncture-Resistance Time (s)			Mucoadhesion Force (N)		
	Blank Film	10 mg CAP Film	20 mg CAP Film	Blank Film	10 mg CAP Film	20 mg CAP Film	Blank Film	10 mg CAP Film	20 mg CAP Film
0	30.4 ± 4	18.8 ± 3.2	14.4 ± 2	10 ± 0.5	11.5 ± 0.4	12 ± 1	8.5 ± 2.2	15.1 ± 2.4	18.5 ± 1
30	20 ± 3.6	6 ± 0.5 *	5.2 ± 0.5 *	5.8 ± 0.9 *	8 ± 0.3 *	8.7 ± 0.9 *	4.6 ± 1.3	7.7 ± 0.9 *	9.2 ± 2.4 *
60	15.6 ± 1.3 *	5.8 ± 0.6 *	4.1 ± 0.4 *	4.5 ± 0.5 *	4.6 ± 0.8 *	3.7 ± 0.7 *	3.5 ± 0.7 *	6.6 ± 2.5 *	6.1 ± 2.2 *
90	15.6 ± 3.8 *	5 ± 0.5 *	3.2 ± 0.4 *	3.4 ± 0.8 *	4 ± 0.7 *	4.3 ± 1.4 *	3.4 ± 0.5 *	5.8 ± 1.4 *	8.2 ± 1.9

**Table 4:** Composition of the polymer film solution.

Sample No.	Chitosan (*w*/*w*%)	Glycerol (*w*/*w*%)	Ascorbic Acid (*w*/*w*%)	Acetic Acid (*w*/*w*%)	Captopril (mg/4 cm^2^)
1	1.4	0.42	2.5	-	-
2	1.4	0.42	2.5	-	10
3	1.4	0.42	2.5	-	20
4	1.4	0.42	-	2.5	-
5	1.4	0.42	-	2.5	10
6	1.4	0.42	-	2.5	20

## Data Availability

Data are contained within the article.

## Abbreviations

The following abbreviations are used in this manuscript:

AA	Ascorbic acid
AC	Apical compartment
ACEI	Angiotensin-converting enzyme inhibitor
API	Active pharmaceutical ingredient
BC	Basolateral compartment
BCS	Biopharmaceutics Classification System
CAP	Captopril
CHI	Chitosan
CHI-Ac	Chitosan acetate
CHI-Asc	Chitosan ascorbate
DoE	Design of Experiments
FT-IR	Fourier transform infrared spectroscopy
GLY	Glycerol
HATR	Horizontal attenuated total reflectance
HBSS	Hank’s balanced salt solution
ICH	International Council for Harmonization
NR	Neutral Red
PB	Phosphate buffer
Ph.Eur.	European Pharmacopeia
QbD	Quality by Design
RH	Relative humidity
SD	Standard deviation
